# The Path to Sustainable Oral Healthcare to Foster Sustainability in Dentistry

**DOI:** 10.1055/s-0044-1785187

**Published:** 2024-05-14

**Authors:** Muhammad Amber Fareed, Muhammad Sohail Zafar

**Affiliations:** 1 Department of Clinical Sciences, College of Dentistry, Centre for Medical and Bio-allied Health Sciences Research, Ajman University, Ajman, United Arab Emirates; 2 Department of Restorative Dentistry, College of Dentistry, Taibah University, Al Madina Al Munawwarrah, Saudi Arabia; 3 Centre of Medical and Bio-allied Health Sciences Research, Ajman University, Ajman, United Arab Emirates; 4 Department of Dental Materials, Islamic International Dental College, Riphah International University, Islamabad, Pakistan

According to the World Health Organization (WHO), 1 climate change weakens essential elements for good health, such as livelihoods, equality, and healthcare access, which disproportionately impact vulnerable groups such as women, children, ethnic minorities, the underprivileged, migrants, and the elderly. Particularly, regions in developing/underdeveloped nations with inadequate health infrastructure struggle most in coping with health issues related to sustainability and climate change and in managing these challenges. 1 The United Arab Emirates hosted the 28th United Nations Framework Convention on Climate Change summit, Conference of Parties Commonly (COP28) in December 2023 and articulated 2023 as the “Year of Sustainability.” 2 In addition to several communities and professions, the healthcare sector also plays a vital role in addressing the challenges posed by climate change being on the frontline of dealing with the direct and indirect health impacts of climate change, including extreme weather events and the spread of diseases.

Furthermore, healthcare facilities and providers must adapt to the changing climate to ensure continued patient care and resilience against climate-related disasters. In terms of mitigation, healthcare operations are energy-intensive and produce greenhouse gas emissions. By adopting energy-efficient practices and transitioning to renewable energy sources, healthcare facilities can reduce their carbon footprints. Healthcare professionals and institutions are also integral in conducting research on climate-related health issues, educating the public on preventative measures, and advocating climate action to protect public health. Beyond this, they can influence policy changes, promote sustainable practices, and set an example by implementing environmentally friendly measures within their own operations.

Dental professionals being an important pillar in the healthcare workforce are integral to the promotion of sustainable dentistry, a practice that actively addresses the intersection of healthcare and climate change. By adopting ecofriendly dental practices, reducing resource consumption, and minimizing waste generation, dental offices can significantly reduce their environmental impact. Working together toward sustainable dentistry will not only facilitate understanding but also foster a comprehensive comprehension among oral health professionals and dental academics about the significance of sustainable dental practices encompassing dental materials, clinical dental procedures, and waste management. Such practices encourage oral healthcare providers to collaboratively identify and outline actionable steps that can be integrated into their daily practices to enhance environmental sustainability, social responsibility, and economic efficiency to embrace and implement sustainable strategies collectively.

The impact of sustainable oral healthcare practices extends beyond dental clinics. Sustainable dentistry also involves reducing the use of harmful chemicals in treatment, benefiting both patient health and the environment. Dental professionals have the opportunity to educate patients about the connections between oral health and overall well-being, including ways in which dental issues can be linked to systemic health problems exacerbated by climate change. They can advocate sustainable healthcare practices and contribute to research on environmentally friendly dental materials and procedures. However, the primary hurdle in adopting sustainability lies in the behaviors and attitudes prevalent within the provision of healthcare in dentistry professionals, who often overlook or do not prioritize sustainable practices. 3

The FDI World Dental Federation (FDI) developed the sustainability in dentistry interactive toolkit to assist dentists and their teams in more sustainable practices and it certainly supports oral healthcare professionals in diminishing their environmental footprint and achieving sustainability. 4 Nonetheless, by incorporating sustainability into education and clinical practices, dental professionals play a pivotal role in mitigating climate change and reducing the environmental footprint of dental care. 5 Similarly, the PRUDENT project (Prioritization, incentives and Resource use for sUstainable DENTistry) proposed to enhance access to essential oral care, aligning with the goal of universal health coverage and providing sustainable implementation strategies for oral health. 6

The WHO Global Oral Health Report 7 serves as a crucial reference for policy-makers, incorporating sustainability dentistry, and offers country-specific profiles to guide decision-makers. This report emphasizes the urgent need for action, revealing alarming global oral health challenges. 7 Therefore, capacity building elements to equip oral health professionals with up-to-date information on sustainable dental practices, materials, and techniques are need to time. 3 4 5 It can be achieved by providing training in evaluating the environmental impact of dental choices and procedures, fostering critical thinking and engaging in the dialog of sustainability dentistry. This will certainly help to encourage a shift in mindset and practices through education, shared goal-setting, and empowering oral health academics to be leaders in promoting sustainable oral healthcare practices within their communities.
